# Early manifestations in a cohort of children prenatally diagnosed with 47,XYY. Role of multidisciplinary counseling for parental guidance and prevention of aggressive behavior

**DOI:** 10.1186/1824-7288-38-52

**Published:** 2012-10-03

**Authors:** Faustina Lalatta, Emanuela Folliero, Ugo Cavallari, Marina Di Segni, Barbara Gentilin, Roberto Fogliani, Donatella Quagliarini, Paola Vizziello, Federico Monti, Luigi Gargantini

**Affiliations:** 1 UOSD di Genetica Medica Fondazione IRCCS Cà Granda Ospedale Maggiore Policlinico, via della Commenda 12, 20122, Milano, Italy; 2 UNITA DI GENETICA CLINICA Fondazione IRCCS Cà Granda Ospedale Maggiore Policlinico, via commenda 12, 20122, Milano, Italy; 3 Servizio di Genetica, Azienda Istituti Ospitalieri, Cremona, Italy; 4 Laboratorio di Genetica Medica IRCCS Cà Granda Ospedale Maggiore Policlinico, via commenda 12, 20122, Milano, Italy; 5 UOS di Diagnosi Prenatale, Clinica Ostetrica e Ginecologica I IRCCS Cà Granda Ospedale Maggiore Policlinico, via della Commenda 12, 20122, Milano, Italy; 6 UONPIA, Fondazione IRCCS Cà Granda Ospedale Maggiore Policlinico, Via Pace 9, 20122, Milano, Italy; 7 UO Pediatria, AO Treviglio, Treviglio (BG), Italy

## Abstract

**Background:**

An increasing number of foetuses are recognized as having double Y because of the widespread use of prenatal screening using chorionic villus sampling and amniocentesis. 47, XYY karyotype occurs in about one out of 1,000 newborn males, but it is not often detected unless it is diagnosed during prenatal testing. Despite the fact that unbiased follow-up studies demonstrate largely normal post-natal development of young men with 47, XYY, there is a scarcity of controlled studies about the neurological, cognitive and behavioural phenotype which remains the main reason for anxiety and anticipatory negative attitudes of parents. Furthermore, prejudices still exist among professionals and the general population concerning the relationship between this sex chromosome aneuploidy and aggressive and antisocial behaviours.

**Methods:**

We report on the clinical follow-up of children diagnosed prenatally with a 47,XYY karyotype, whose parents received multidisciplinary counselling and support at time of diagnosis. The specific focus of our study is on auxology, facial features, developmental milestones, behaviour, detection of aggressiveness as well as the evaluation of parental attitudes toward prenatal counselling. Clinical evaluations including auxological measurements and dysmorphological descriptions were as conducted on 13 boys aged 9 month -7 years. The Child Behavior Check List test specific for age and a 15 item questionnaire were administered to both parents. An update of ongoing problems was carried out by means of a telephone interview two years later.

**Results:**

Our results show that, from birth, weight, height and head circumference were above average values while some facial features such mild hypertelorism are overrepresented when compared to parents' facial features. Language delay was detected in 8 out of 11 children older than 20 months. Parental attitudes were found to be favourable toward prenatal diagnoses of sexual chromosome aneuploidies.

**Conclusions:**

Our data, although limited, is similar to other observational studies, and serves to alert clinicians about opportunities to delineate new and appropriate educational interventions that target the specific learning challenges of XYY boys. Our experience better defines the early manifestation of XYY and should aid those involved in prenatal counselling and paediatric surveillance.

## Background

47, XYY karyotype is an aneuploidy of the sex chromosomes in which a human male receives an extra Y chromosome. It occurs in about one out of 1,000 newborn males, but it is not often detected unless it is diagnosed during prenatal testing. An increasing number of foetuses are recognized as having double Y because of the widespread use of screening using chorionic villus sampling and amniocentesis. A prenatal identification of a 47, XYY male signals the need for a complex and challenging genetic counselling whose outcomes have not yet been fully explored. The discovery of such an anomaly during pregnancy always involves a great deal of anxiety and presents a dilemma for the prospective parents who need to understand both the clinical consequences and to understand the impact of an unexpected genetic condition on their child’s quality of life and on their relation to their son. Despite the fact that unbiased follow-up studies demonstrate largely normal post-natal development of young men with 47, XYY, characterized primarily by tall stature [1], there is a scarcity of controlled studies about the neurological, cognitive and behavioural phenotype which remains the main reason for anxiety and anticipatory negative attitudes [1-11]. Furthermore, prejudices still exist among professionals and the general population concerning the relationship between this sex chromosome aneuploidy and aggressive and antisocial behaviours. This is mostly related to studies conducted during the 1970s which seemed to show an increased frequency of 47, XYY males among people who have entered the criminal justice system [6,8]. Web based queries about 47, XYY are packed with statements and descriptions about the genetic determinism of aggressive temperaments. Therefore, parents, looking on their own for genotype-phenotype correlations from uncontrolled, and possibly inaccurate, sources are made more anxious. The same can be said for poorly informed professionals who offer genetic counselling after the cytogenetic diagnosis. Such misinformation might lead to an unwarranted pregnancy termination or to a difficult relationship between mother and child. Integrated counselling with the shared competences of a clinical geneticist, who deals with the origin of chromosomal abnormalities and genotype-phenotype correlations; a paediatrician who is experienced with the health problems of 47,XYY children and who understands the specific areas of difficulty such as language delay; and a psychologist who can assist the perspective parents deal with the decision making process is an approach still confined to large facilities such as university medical centers. The aim of the multidisciplinary approach is to supply parents with sufficient knowledge and confidence to empower them to deal with the uncertainties that surely follow the diagnosis. Over the last ten years we have set up such a team-based approach to manage pre- and postnatal counselling so as to make best use of expert guidance and give parents a balanced view what they may expect in infancy and adulthood of their 47, XYY male children. a considerable effort has been made to homogenize the contents of various step to avoid conflicting messages.

Parents has never been forced but, according to individual needs they request the other specialist advice after first communication by the clinical geneticist. Progression toward awareness is an individual path.

In this study we present the outcomes of 18 cases of 47, XYY children followed after birth whose parents agreed to continue the pregnancy after prenatal detection of the aneuploidy. Couples gave their consent to be recalled, to take part in a clinical follow up and to be interviewed and to respond to the Child Behaviour Checklist (CBCL). CBCL is a device by which parents can rate the child’s problem behaviors and competencies.

## Patients and methods

Between January 2005 and December 2008, 21 Italian couples were referred to our Clinical Genetic Unit in Fondazione IRCCS Cà Granda Ospedale Maggiore Policlinico, in Milan, for genetic counselling following a prenatal diagnosis of 47, XYY that had been performed in the first or second trimester of pregnancy.

All couples in which the prenatal diagnosis was performed had received a non-disclosing phone-call at the time of diagnosis and were offered genetic counselling within 24 hours. All counselling were offered by a single clinical geneticist (F.L.) and was then followed by a second session with a paediatrician with specific expertise in the clinical management of childhood sex chromosome aneuploidies (L.G.). Finally all couples were offered psychological counselling (D.Q.) to support their decision concerning the pregnancy and to help them deal with anxiety and uncertainty (Table 1).

**Table 1:** Main topics discussed by the different specialists during the prenatal counselling session

**Counselling session, specialist**	**Main topics**
Clinical geneticist	Karyotype description, origin of chromosomal abnormalities, accuracy of prenatal diagnosis, genotype-phenotype correlation,
Pediatrician	Clinical consequences of aneuploidy, natural history of affected boys, prevalence and severity of symptoms, diagnostic assessment and reference centres for complicated cases and therapeutic facilities,
Clinical Psychologist	Support aimed at dealing with psychological implications of diagnosis and prognosis Specific advice to help parents integrate their own histories into the development of coping skills and behaviours and to encourage resilience.

Afterwards all couples reported using a “web based” interface; behavioural disturbances and aggressiveness were frequently discussed.

Eighteen couples decided to continue the pregnancy while 3 couples requested termination. All parents who accepted childbirth were counselled against disclosure of the genetic anomalies to relatives and paediatricians for the first year of child life.

In June 2009, the 18 couples were contacted by telephone and invited to take part in a survey which included a clinical assessment of the child, an interview and a questionnaire. Among these, 13 cases accepted and were included in our study and 2 couples could not be found. Three couples agreed only to participate in a phone-based questionnaire due to excessive distance from our Centre and were therefore excluded from the study.

Parents’ medical history and social data were collected.

A physical assessment of the child was performed by a clinical geneticist and a paediatrician: it included a detailed personal history, a physical evaluation, auxological measurements, description of minor anomalies of face and developmental milestones with specific attention to language delay. With parents' consent, pictures of the child were taken to document dysmorphisms and familial traits.

The interview with parents was carried out by clinical psychologists and included 15 questions aiming at evaluating the relationship with the paediatrician; how couples dealt with the prenatal diagnosis and genetic counselling; and how they coped with specific circumstances of the prenatal and postnatal period. Special attention was given to a retrospective judgment about prenatal communication, present and future worries, needs and expectations.

Information about the children’s competencies and behavioural/emotional problems were collected with the Child Behaviour Checklist (CBCL) for ages 2–3 and 4-18 [2]. The CBCL is one of the most commonly used scales of infantile behavior in Italy and in other countries and it is employed for both clinical and research purposes. The CBCL is an indirect multidimensional questionnaire, filled in by the parents, which explores the child’s behavioral and emotional repertoire and allows one to investigate both social abilities and behavior disturbances. Following our protocol, both parents together answered a multiple choice questionnaire that used a three-valued scale which identified the frequency of specific behaviors. The data-base allowed us to draw a personalized profile for each child aimed at revealing difficulties in the investigated areas.

Clinical qualitative traits and interviews data were evaluated by descriptive analysis and the CBCL test data were compared to those derived from an Italian population matched for age.

Data derived from this instrument allowed us to describe the following: involvement in activities (sports and spare time), emotional response (anxiety, hyperactivity, etc.) and behavior (somatization, withdrawal).

This procedure allows emotional disturbances to be classified as either internalizing disturbances (emotional hyper control) as opposed to externalizing disturbances (emotional control deficit). Answers to the items are grouped in 8 categories: anxiety and depression; withdrawal; somatic complaints; social problems; cognitive problems; attention problems; transgressive behaviour with the breaking of rules; and aggressive behaviour.

Furthermore behaviour can be evaluated according to six scales, based on the DMS-IV classification: affective problems, anxiety problems, somatic problems, attention problems, oppositive -provocative behaviour and conduct problems.

In June 2011, two years after the first evaluation, the same couples received a phone call from the clinical geneticist, to review items previously discussed and to inquire about specific actions taken to overcome difficulties. All couples consented to the phone call and the following data were collected: health problems during the previous two years, social difficulties at school and with peers, presence of language delay and action taken to increase language skills.

## Results

### Auxological, social and developmental milestones data

Our cohort includes 13 pairs of parents with children diagnosed prenatally with a 47,XYY chromosome. In fact, 14 children were seen, including one pair of male twins among whom only one has this genetic characteristic; but their parents did not have the karyotype repeated after birth so as to avoid their own prejudices in dealing with growth and development.

**Figure 1:**
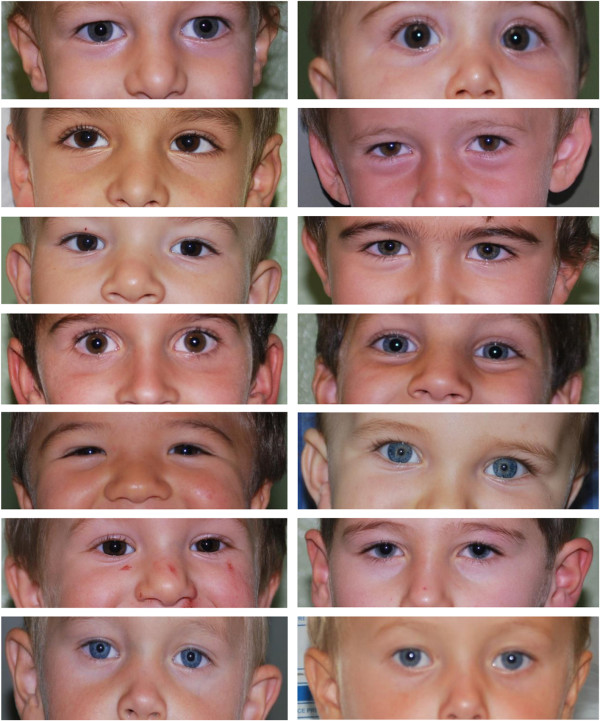
**Facial features.** Orbital and malar region of 47,XYY probands showing mild hypertelorism, broad nasal bridge and mild flat malar region. **A** and **B** Couple of dizygotic males twins. One with 47,XYY prenatal diagnosis.

The age of the children was 9 months to 7 years: 7 cases were between 9 months and 3 years, 6 between 3 and 7 years. The mean maternal and paternal age at birth was 39 and 42 years, respectively. Eight couples out of 13 were in their first pregnancy. Fifty % of parents were graduates, 50% had a high school certificate. No congenital anomalies were observed. 11 children were reported as healthy, 3 presented with medical problems including recurrent bronchospasm, epilepsy and infantile pubarche. At the two year follow-up no relevant health problems were reported. Table 2 summarizes auxological data at birth and main developmental milestones while auxological data at evaluation are reported in Table 3. No evident dysmorphism was noted at clinical evaluation. Frequent facial features were mild hypertelorism, broad nasal bridge, low-set ears and mild flat malar region. These characteristics were confronted with that of parents and were described as specific of children (Figure 1). Language skills were specifically asked about and, considering the age range 33 – 108 months, all children but one had acquired some language. Four out of 12 demonstrated a limited evolution of language. Parents of 5 of the affected children had inquired about specific speech and language therapy.

**Table 2:** Summarizes auxological data at birth and main developmental milestones

	**Mean value**	**Min. – Max.**
Weight at birth (g)	3089	2030-4385
Length at birth (cm)	49,6	45-53
Occipital-frontal circumference at birth (cm)	34	32-36
Age at first words (months)	22	12-42
Age first walking (months)	13	10-17
Age at evaluation (months)	40	9-82

**Table 3:** Auxological evaluation table

**Case**	**Age**	**Weight**	**Height**	**HC**	**ICD**	**OCD**	**AS**	**Palm**	**Middle finger**
		**kg**	**cm**	**cm**	**cm**	**cm**	**cm**	**cm**	**Cm**
1	00y 09 m	11,6 (90–97)	80,0 (>97)	49,0 (>97)	3,0 (>97)	8,6 (>97)	NA	6 (>97)	4 (75–97)
2a*	02y 06 m	13,0 (25–50)	93,5 (50–75)	50,6 (50–75)	2,8 (50–75)	8,0 (75–97)	NA	6,2 (25–50)	4,5 (25–50)
2b*	02y 06 m	14,3 (50–75)	92,0 (25–50)	51,0 (75–90)	3,0 (75–97)	8,0 (75–97)	NA	6,2 (25–50)	4,5 (25–50)
3	04y 01 m	18,0 (50–75)	106,5 (50–75)	52,5 (75–90)	2,8 (50–75)	8,0 (50–75)	NA	7 (50–75)	5 (25–50)
4	02y 06 m	15,0 (50–75)	97,6 (75–90)	50,5 (50–75)	2,3 (3–25)	8,3 (75–97)	98	6,6 (50–75)	4,5 (25–50)
5	02y 01 m	15,0 (75–90)	92,5 (75–90)	50,3 (75–90)	3,4 (>97)	8,6 (>97)	94	7 (75–97)	4,8 (75–97)
6	02y 10 m	15,0 (50–75)	97,2 (50–75)	51,0 (50–75)	2,5 (25–50)	8,0 (75–97)	100	6,8 (75–97)	4,4 (50–75)
7	00y 11 m	10,1 (50–75)	72 (10–25)	NA	2,6 (50–75)	7,3 (50–75)	NA	4,8 (3–25)	3,5 (25–50)
8	05y 02 m	21,8 (50–75)	116 (75–90)	52,5 (50–75)	2,6 (25–50)	8,2 (50–75)	NA	7,5 (50–75)	5,2 (25–50)
9	05y 04 m	23,5 (75–90)	120 (10–25)	53 (75–90)	3,0 (50–75)	8,5 (75–97)	118	7,6 (50–75)	5,5 (50–75)
10	06y 10 m	24,8 (50–75)	124 (50–75)	53 (50–75)	2,8 (50–75)	8,8 (75–97)	NA	7,8 (50–75)	6,0 (50–75)
11	02y 00 m	16,0 (90–97)	89 (50–75)	52 (90–97)	2,8 (75–97)	8,0 (75–97)	88	6,5 (50–75)	4,0 (3–25)
12	04y 07 m	18,0 (25–50)	105 (10–25)	51 (25–50)	3,0 (50–75)	9,0 (>97)	108	7,0 (25–50)	5,0 (25–50)
13	05y 00 m	19,0 (25–50)	NA	48,5 (<3)	2,7 (25–50)	8,0 (50–75)	107	7,0 (25–50)	5,2 (25–50)

* Cases 2a and 2b are dizygotic twins and only one of them has a 47,XYY karyotype.

*HC* = Head circumference, *ICD* = Inner canthal distance, *OCD* = Outer canthal distance, *AS* = Arm span. *NA* = Not available. Percentiles derived from auxological curves of Italian (if available) or international values of pediatric cohorts are reported in brackets.

**Weight and Height percentiles >2 years:** Cacciari, Milani, Balsamo & Directive council of SIEDP/ISPED for 1996–97 and 2002–03 J Endocrijnol Invest 29(7):581–593, 2006.

**Weight and Height percentiles <2 years:** Longitudinal Growth Studies, London Group.

**Head Circumference:** Longitudinal Growth Studies, London Group.

**Inner Canthal Distance, Outer Canthal Distance, Philtrum Lenght, Palm Lenght, Middle Finger Lenght:** Feingold and Bossert. Birth Defect. Orig Art Series 10(13), 1974.

### Relationship with pediatrician

In our sample 8 couples out of 13 did not disclose the cytogenetic diagnosis to their paediatrician, following the advice given at the time of prenatal diagnosis by the clinical geneticist. The remaining 5 couples preferred to inform him "*by choice*", "*to be correct*" or, in 1 case due to a clinical problem (the early growth of pubic hair). According to parents, none of the paediatricians who were informed of the 47,XYY karyotype gave any specific advice.

### Interview with parents

Table 4 summarizes the items of parent’s interview.

**Table 4:** Results from the interview with parents

**Questions**	**Not at all**	**Not very much**	**Enough**	**Somewhat**	**Very much**
**Do you think that prenatal diagnosis was useful in your case?**	0	1	2	6	4
**Were you worried at karyotype communication?**	0	2	1	2	8
**Were you worried after genetic counseling?**	3	6	2	0	0
**Do you think that provided information was adequate?**	0	0	2	6	5
**Do you think that genetic counseling supported you psychologically?**	0	0	1	7	5
**Do you remember information received in genetic counseling?**	0	0	1	5	7
**Do you perceive differences between your son and the same age group?**	7	4	1	1	0
**Do you perceive differences between your son and siblings? (7 are only children)**	2	0	3	0	1
**Does your son communicate with relatives?**	0	0	0	3	10
**Does your son communicate with strangers?**	0	1	4	4	4
**Does your son participate in play activity with other children?**	0	1	3	3	6
**Are there any aspects in your child’s behavior that arouse in you some concern?**	9	1	1	1	1
**Are you interested in future updates regarding 47,XYY karyotype?**	0	0	2	2	9
**Have you have the desire to obtain further expert opinions?**	12	1	0	0	0
**In the past few years had you wished to have more information?**	6	5	1	1	0

All interviewed parents but one considered prenatal cytogenetic test very useful. Reaction to fetal karyotype communication was in most cases alarming, including states of uncertainty (1 case), worry (2 cases) and trauma (8 cases), only two cases reported little concern.

An explanation of foetal karyotype with an improved understanding allowed couples to regain some stability. In 3 couples out of 13 their anxiety was almost completely mitigated; residual apprehension was present in 6 cases; a slightly increased level of unease was felt in only 2 cases.

All parents rated the content and adequacy of information received from the team as very positive; sometimes genetic counselling even supplied emotional support, which was considered “good” (8 cases) or “very good” (5 cases).

At the time of interview all parents clearly remembered the clinical information received.

Seven couples of 13 didn’t perceive any differences between the affected child and same age peers. Four, children aged between 4 and 5 years, perceived some irrelevant differences. In the six families in which siblings were present, parents perceived greater differences between their affected and unaffected children especially more shyness and language delay.

The ability to participate in play activities was reported as normal in all cases. In 2 children were described as "observers", "watching", "they needed to become familiar”. Some of the traits recorded were similar to individual differences present in the same general age-matched population.

For most parents their son’s future was not worrying (9 cases), or it caused little concern (2 cases), while some doubts and fears remained in a few couples (2 cases). Almost all parents did not feel the need to consult other medical specialists. In only one case (a 5 year-old child) speech therapy was undertaken. However half of couples had sought more information on the Internet. The couples interviewed declared a strong, specific interest on future developments in genetic research on boys with a 47,XYY karyotype (9 cases).

### CBCL

The quantitative analysis of data obtained through administration of the CBCL 2–3 to 5 families demonstrated in 3 of them the almost complete absence of pathological aspects related to behavior, internalizing problems (anxiety, depression, withdrawal, inhibition, shyness, low self-esteem), and or externalizing problems (aggressive behavior, disobedience to rules, intolerance to frustrations). In the other two cases, although their scores fall within the normal range, a non-homogeneous profile, specifically with respect to externalizing behavior and sleeping, has been identified.

Administration of CBCL 4–18 to 6 families, revealed a normal profile in 4 of them. One child with clinical profile was perceived by his parents as disadvantaged in his relationship with peers and adults, showing shyness, withdrawn behavior and difficulties in relationships towards strangers. In particular the two higher scores regarded social problems (70) and internalizing problems (66). The other child was perceived as exhibiting poorer average socialization, aggressive behavior, irritable temper, and with difficulties in dealing with rules at home and at school because of inattention and frustration in the relationships with his peers. In particular the higher scores are aggressive behavior (69) and externalizing problems (66).

In summary, aggressive temperament and behavior were reported in 2 cases out of 11, with both children being > 5 years of age. In the remaining cases the profiles did not reveal any pathological traits or clinically relevant discomfort in all investigated area.

## Discussion

Over the last few decades there has been a significant increase in the number of XYY males detected prenatally which now represents about 10% of all cases according to Visootsak et al. [12]. In order to properly educate parents during pregnancy and to provide adequate surveillance later on, it is of utmost importance to obtain more data on the developmental profiles of boys with a karyotype 47, XYY and on potential problem areas during later development.

A very recent systematic review [13], in fact, revealed that studies conducted in the last 20 or 30 years do not contribute much to the understanding of the real clinical impact of the extra Y chromosome [5] This is probably due to a small and often biased cohort, an inhomogeneous selection of testing modalities and an absence of longitudinal follow-up, a deficiency which prompted Legget et al. to exclude 656 out of 702 articles identified by their literature search. Despite the huge analysis and selection, the data presented by the above authors are not conclusive with respect to the long term neurocognitive prognosis of 47, XYY boys [14].

Our present study deals with a small cohort of children prenatally recognized as 47, XYY, which is unique in that care was taken so that the style and content of the information given to parents immediately following prenatal testing was as uniform as possible. Disclosure of chromosomal aneuploidy and subsequent counseling sessions were homogeneous and parents were given the chance to report their worries generated by unassisted web queries. Couples were then contacted at different intervals and invited to a clinical follow-up with the aim of collecting data about health problems, developmental milestones and parental adjustment to the diagnosis.

Our results show that all boys were normal in appearance. Nevertheless, we suggest that a somewhat specific facial phenotype is present particularly involving the ocular and malar region. We frequently noted mild hypertelorism, broad nasal bridge, low set ears and mild flat malar region. Measured values for inner and outer canthal distance confirmed the tendency to a mild hypertelorism as we previously had observed in 43 females with XXX [15].

Growth and health data are similar to that reported in the literature [3]. Specifically, weight, height and head circumference values were above average in 10 out of 12, 8 out of 11 and 9 out of 11 children, respectively (not including twins and cases with missing data). It has been proposed that tall stature, generally present in all sex chromosome polysomies, is related to the overexpression of growth-related genes, including SHOX [11], on the X and Y chromosomes.

Conversely an above average head circumference is not reported in other sex chromosome aneuploidies and may be an exclusive characteristic of Y chromosome polysomies due to a still undefined mechanism.

To establish the prevalence of language delay we reinter viewed the parents at a time when most of the boys should have been speaking (33–108 months). Mothers, already alerted to the relevance of possible language delay, answering a series of questions, reported inadequate language and speech development in 4 out of 12 cases. Two boys, affected by a more persistent language disorder, were sent for specific language and speech therapy.

In our population of boys 4–11 years old, in particular, the severe delay of verbal production appears to be greater, both in oral and written form [3,9,13].

Distractibility, which appears to be a feature of these children, emerges between 2 and 3 years as a prognostic sign of a possible future learning disability and an attention or hyperactivity disorder. The behavioral phenotype described in the literature suggested that the language delay was a determining factor in these patients with IQs in the lower range of normal. Distractibility or attention deficit disorder are commonly noted in the literature, and said to be present in 82% of boys with XYY, often interfering with academic performance [3,12]. The direct consequences of this are likely to be a decrease in the level of academic performance and an increase in aggressive behavior, possibly even anticipating a real shake-reactive anti-social as a reaction to severe frustration.

Indeed, the frequency of behavior disorder and depressive reactions to stressful events was found to be greater in 47, XYY patients compared to a control group of 46, XY boys [12,15].

The profiles obtained by administration of the CBCL to parents of our 11 patients are quite comparable to data reported in the most recent literature. Because we felt that such findings needed further explication we decided to employ the CBCL questionnaire which evaluates the parents’ assessments of their children’s development. The results proved problematic in some areas in only 2 cases. Qualitatively, the clinical manifestations arose from a difficulty in exercising appropriate emotional controls (internalizing problems, and difficulty in relationship) or from a lack of any control (externalizing problems, aggressive acting out). Specifically, the problems identified related to the area of social relations.

In the case of the first child closure and emotional difficulties seem to prevent adequate socialization. The answers given by the child's parents emphasize his isolation, especially from his peers: the child is, however, currently being treated in the Department of Psychiatry. Considering the possible evolution of these patients to schizotypal and psychotic disorders in adulthood the particular relationship difficulties in this patient might suggest a pervasive developmental disorder. In the second case the child’s aggressive behavior affected his peer relationships: this picture seems more in line with what has been generally reported and was preceded by a language disorder (stuttering).

Excessive parental attention and desire to control their child’s temper could explain the higher level of control and worries about the child’s actions and their judgment about the quality of aggressiveness.

Other areas probed by the CBCL such as internalizing and externalizing problems were found mostly to be within the normal range. Given these observations it seems important to enable appropriate and timely educational intervention involving parents and schools targeted to specific learning goals [12]. At the same time an overall assessment would allow for the definition of an educational-behavioral treatment plan and possibly rehabilitation. At present there is little data to support the efficacy of this approach [5].

The reported temperamental traits might be explained within the interindividual variability, related to the context of its development. Given these observations it seems important to enable appropriate and timely educational interventions involving parents and school, targeted to specific learning goals [12]. The main limit of our study is the lack of a control group. We consider this an unavoidable limitation because a well designed control group would have been impossible to define. According to our own observations and based on our experience with multidisciplinary prenatal counselling, we propose active post-natal monitoring for those parents who decide to proceed with a pregnancy after the detection of 47,XYY. The parents themselves raised the question of the early detection of potential diversity, facilitating the control of anxiety and providing them with suggestions to avoid contact with inexperienced specialists. We suggest that there be two clinical evaluations each year in the first three years to reveal any early indicators which would prompt immediate intervention [7,12].

## Conclusion

Prenatal detection of a chromosome abnormality, no matter what its clinical implications, is always very traumatic and can be devastating to a couple if they are forced to make a decision by themselves without assistance or counseling.

Although sex chromosome aneuploidies were first described many decades ago [16], it is only within the last few years that large-scale and well-controlled studies are documenting detailed physical and cognitive profiles of the disorders. Most recently several investigators have demonstrated an increase risk for neurodevelopmental disorders, especially those affecting language; but many children do develop normally. It is therefore of utmost importance that parents receive good multidisciplinary counseling at time of prenatal diagnosis, and be informed as follows which should allow for a conscious, hopefully more rational choice regarding the pregnancy:

– Children with 47,XYY do not have specific needs for care and can be followed by a pediatrician who should treat them similarly to the normal pediatric population.

– There must be specific attention to language development by the parents and the physician in order to detect delay or difficulty of expression.

– In case of an abnormal neuropsychological profile, there needs to be directed support and early follow-up to minimize possible detrimental effects of language and communication delay. Its availability would be guaranteed by the multidisciplinary team which had been put in place before the birth of the child and might help to reduce aggressiveness related to frustration and low self esteem.

At the moment very few Prenatal Units have put together a multidisciplinary path to bridge the gap between the pre- and post-natal periods. We believe that his model should be promoted.

## Competing interests

The authors declare that they have no competing interests.

## Authors’ contributions

FL, RF, DQ and LG are the component of the prenatal team which takes care of procedures, counselling and support. MD did the cytogenetic diagnosis; EF is the psychologist who organized the follow-up, UC and BG did auxological measurements and data-base, FM e PV took care of parents’ interview and CBCL test. All authors read and approved the final manuscript.
